# Parallel processes of temporal control in the supplementary motor area and the frontoparietal circuit

**DOI:** 10.1002/pchj.701

**Published:** 2023-12-17

**Authors:** Xuanyu Wang, Shunyu Shi, Yan Bao

**Affiliations:** ^1^ School of Psychological and Cognitive Sciences Peking University Beijing China; ^2^ Graduate School of Systemic Neurosciences Ludwig‐Maximilians‐Universität München Munich Germany; ^3^ Institute of Medical Psychology, Ludwig‐Maximilians‐Universität München Munich Germany; ^4^ Beijing Key Laboratory of Behavior and Mental Health Peking University Beijing China

**Keywords:** duration reproduction, fMRI, neural mechanism, time perception

## Abstract

Durations in the several seconds' range are cognitively accessible during active timing. Functional neuroimaging studies suggest the engagement of the basal ganglia (BG) and supplementary motor area (SMA). However, their functional relevance and arrangement remain unclear because non‐timing cognitive processes temporally coincide with the active timing. To examine the potential contamination by parallel processes, we introduced a sensory control and a motor control to the duration‐reproduction task. By comparing their hemodynamic functions, we decomposed the neural activities in multiple brain loci linked to different cognitive processes. Our results show a dissociation of two cortical neural circuits: the SMA for both active timing and motor preparation, followed by a prefrontal–parietal circuit related to duration working memory. We argue that these cortical processes represent duration as the content but at different levels of abstraction, while the subcortical structures, including the BG and thalamus, provide the logistic basis of timing by coordinating the temporal framework across brain structures.

## INTRODUCTION

Timing is a crucial aspect of sensory processing and motor planning in the brain. Durations in the range of seconds to minutes are relevant for cognitively controlled behavior, requiring active mental engagement. With the help of functional imaging techniques, researchers have found timing‐associated activities across multiple brain regions, including subcortical structures in the basal ganglia (BG; Ferrandez et al., 2003; Rao et al., 2001); cortical structures, such as the supplementary motor area (SMA) (Kotz et al., 2016; Protopapa et al., 2019; Teki et al., 2011); the pre‐SMA (Coull & Nobre, 2008; Harrington et al., 2011; Rao et al., 2001); the insula (Kosillo & Smith, 2010; Wittmann et al., 2010); and the cerebellum (Pfeuty et al., 2015; Rao et al., 2001). However, the functional role of these regions and their inter‐regional organization remain unclear (Pöppel, 2004; Zhou et al., 2014). One difficulty of conducting neuroimaging studies for timing tasks presumably comes from parallel cognitive processes that are hard to temporally disentangle.

The accumulation of the blood‐oxygen‐level‐dependent (BOLD) signal has been taken as the neural substrate of temporal encoding in many studies (Casini & Vidal, 2011; Wittmann et al., 2010), thus treating the encoding of duration as an accumulation process, similar to other decision‐making tasks (Balcı & Simen, 2016; Wittmann, 2013). However, no physiological evidence on the neuronal or circuit level has been established in support of the link between climbing hemodynamic signal and accumulation of neural information. More importantly, it is unclear whether the climbing signal is a result of persistent non‐temporal processes or the dedicated timing.

In timing tasks, “time” is at the same time the logistic, contextual framework, and the content of the neurocognitive processes (Pöppel, 1997). Therefore, it is hard to tell from the data whether the temporally evolving neural activity is simply a byproduct of persisting non‐timing parallel processes that last the same period being timed. For example, the duration‐reproduction task is popular in timing research partially because of its complex cognitive spectrum (Maaß et al., 2019; Shi et al., 2013). In a typical scenario, subjects are first presented with an external event that lasts for a while, the duration of which is the sample to be encoded and memorized. Following a delay, the subjects perform the anticipatory timing based on the reference memory of this sample duration (i.e., to reproduce the remembered duration; Mioni et al., 2014). During encoding, the internal cognitive process of timing shares identical time course with the external presentation of stimuli, thus temporally collides with sensory processing and other task‐general components, like elevated attention. For reproduction, the motor timing is similarly contaminated by sensory processing, attention, or motor preparation.

One physiological model of timing is the BG–SMA circuit (Kotz et al., 2016; Wittmann et al., 2010). The BG, especially the putamen, is considered as the subcortical clock governed by midbrain dopaminergic inputs and synchronizes the cortical processes across multiple regions (Cheng et al., 2016; Meck, 2005). This is supported by pharmacological interference of timing by dopamine antagonists and agonists (Fung et al., 2021; Parker et al., 2015), as well as clinical evidence with dopamine‐deficit patients (Högl et al., 2014; Jones et al., 2008). The BG–SMA theory proposes that the BG generates relatively stable‐paced pulses, which are accumulated in dedicated cortical structures, such as the SMA, into a cortical representation of passed time. Based on these assumptions, hemodynamic changes in the BG structures and SMA during timing tasks are often attributed to timing without closer inspection. However, considering the more general role that the BG–SMA circuit plays in goal‐directed, task‐context‐sensitive behavior (Haber, 2003; Korb et al., 2017), it is questionable whether the hemodynamic elevations in functional imaging studies are relevant for active timing, or are simply artifacts of non‐timing parallel processes (e.g. attention and motor preparation). Since an explicit motor response is required in all timing tasks, the preparation for the response is always concurrent with the active timing, thus indistinguishable in the data. This possible confounding has not been considered in previous studies.

One way to disentangle intermixed components is to perform subtraction with control conditions containing only the non‐timing elements. We introduced a motor control and a sensory control to the duration‐reproduction task: a motor control requiring simple reaction and a sensory control with only passive viewing. By comparing the time course of BOLD signals, we were able to decompose neural activities in the brain linked to different cognitive processes. We confirmed that the SMA is bifunctional, participating in both motor planning and timing, with an additive hemodynamic response when both components are present. The fluctuation of hemodynamic signals in subcortical structures, including the putamen and thalamus, are less specific and more likely motion‐related. Working‐memory associated activities were observed in the left dorsal lateral prefrontal cortex and the bilateral intraparietal sulcus. Activity in the SMA leads the frontoparietal circuit during encoding of the temporal duration, while the sequence is reversed during reproduction. Our results dissociate the timing mechanisms into an encoding stage in the SMA and a following memory stage in the frontoparietal circuit.

## METHOD

### Duration‐reproduction task

**FIGURE 1:**
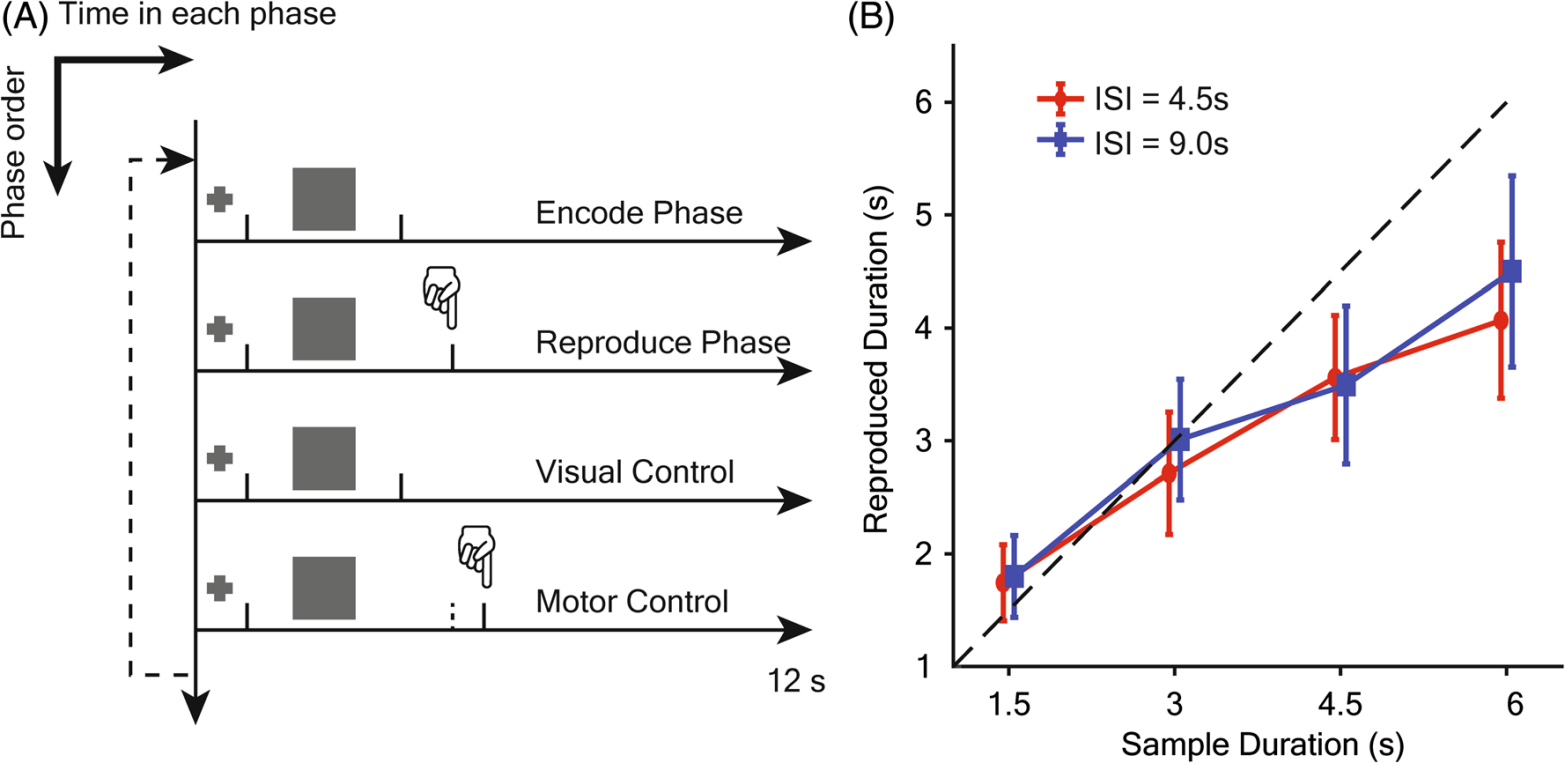
Duration‐reproduction paradigm and behavioral results. (A) Schematic diagram of the duration‐reproduction paradigm during fMRI scanning. In each trial, four phases were in a continuous sequence with a fixed order, durations of all four phases were extended to 12 s. In each phase, a box appeared at the screen center following a visual cue of 1 s. In the encoding and visual control, the visual stimuli persisted for 3 s, while in the other two phases the duration was dependent on the subject‐reproduced duration in that trial. (B) Reproduced duration for each sample duration in two different interstimulus interval (ISI) conditions. Error bar marks the range of 95% confidence interval. No significant difference in reproduction performance was observed between different ISIs. Reproduction for the 3‐s sample duration was more accurate than the over‐reproduced 4.5 s and 6 s.

The whole experiment was divided into five imaging sessions, each with 12 trials (60 trials for each subject). Each trial was made up of four sequential phases: Encoding Phase (EP), Reproduction Phase (RP), Visual Control (VC), and Motor Control (MC), as depicted in Figure 1A. At the beginning of each phase, a visual cue (Size: 0.8° × 0.8° visual angle) appeared for 1 s in the center over a dark background, followed by a square target (Size: 10° × 10° visual angle). In the encoding phase (EP), the square lasted for exactly 3 s as the sample duration. The sample duration was selected based on previous research showing maximum accuracy of reproduction between 3.0 s and 3.5 s (Pöppel, 1972).

In the next phase of the task (RP), participants were asked to reproduce the sample duration by terminating a comparison stimulus with a keypress. In the third phase (VC), the same square was presented again for 3 s and only passive viewing was required. In the last phase (MC), the square was presented for the same duration as reproduced in RP. To control for timing‐irrelevant brain activities (e.g., motion preparation and initiation), participants were asked to make a keypress following the disappearance of the stimulus as soon as possible. They were instructed not to deliberately time the stimuli in the MC phase but only react when the square was gone. In case participants failed to press a key in RP, the stimulus was presented for 11 s and then turned off; the missing trial would be excluded from the analysis. Since temporal processing was previously shown to be more right‐lateralized (Rao et al., 2001; Wittmann, 2013), all key presses were performed with the left index finger.

Each task phase was extended to 12 s with a blank screen, allowing hemodynamic response back to baseline. Identical lengths for each task phase allow us to obtain trial‐average hemodynamic curves for each region of interest (ROI). Trials were firstly screened by the reproduced duration during the RP phase to ensure that participants were attending and following the instructions; further screening was conducted on the reaction time in MC, ensuring no anticipation or active timing. Outlier trials were both defined individually as beyond 2 standard deviations from the mean. This post‐hoc data screening supplements the verbal instructions to guarantee that all trials analyzed were performed as instructed.

### Behavior verification of duration‐reproduction task

Six right‐handed college students (all males; mean age: 21.5 years; age range: 21–23 years) participated in the pilot behavioral experiment, all with normal or corrected‐to‐normal vision and all reporting no neurological, psychiatric, or medical problems.

The experiment was conducted in a soundproofed chamber equipped with a computer. The visual parameters were identical to the fMRI study. Sample durations were randomly chosen from 1.5 s, 3.0 s, 4.5 s, or 6.0 s as the within‐block variable; each of the four alternatives was repeated four times, resulting in 16 trials per block. The interstimulus interval (ISI) following the reference stimuli was either 4.5 s or 9.0 s, randomized between blocks; each ISI was repeated three times, composing six blocks. Participants were instructed to terminate the second visual object with a press on a 4‐key reaction box (Sinorad Inc., China) when its duration matched the sample, and not to apply any timing strategy, such as counting or tapping. Each trial was followed by a 7‐s inter‐trial interval with a blank screen.

All visual stimuli were displayed in the center of a 27‐in. ViewSonic VX2268wm LCD monitor, 80 cm away from the participant. Stimuli display and response collection were performed with the help of Psychtoolbox extensions (Brainard & Vision, 1997) in MATLAB 2014b (MathWorks Inc., USA).

### MRI participants

Sixteen college students (eight females and eight males) were paid for participation in the functional imaging study, with ages ranging from 19 to 28 years (*M* = 21.7 years, *SD* = 2.6). All participants were right‐handed and with normal or corrected‐to‐normal vision. No record of neurological, medical, visual, or memory issues was reported. Participants were given written consent and several practice trials to familiarize themselves with the task before the scanning session. They were asked to maintain a regular daily schedule and not to consume any psychostimulants, drugs, or alcohol within 3 days before the experiment. One subject was excluded from data analysis due to bad signal spatial alignment due to excessive head movement (>4 mm).

### MRI acquisition and preprocessing

MRI data were collected on a 3 T MR750 General Electrical scanner with a 32‐channel head coil. T1‐weighted anatomical images were acquired in 188 slices (3D MPRAGE; 1 × 1 × 1 mm^3^ resolution; gap = 0 mm). Functional T2*‐weighted echoplanar images were acquired with 33 axial slices in interleaved order (3.5 mm thickness, 3.5 × 3.5 mm in‐plane pixel size, echo time = 30 ms; repetition time (TR) = 2000 ms; field of contrast = 224 × 224 mm^2^; matrix = 64 × 64; flip angle = 12°; gap = 0.7 mm). The trial presentation was synchronized to TR onset by scanner trigger pulses. Visual stimuli were back‐projected on a 25 × 19‐cm translucent screen 90 cm away from the participant. Cushions were placed around the head and neck to maximize comfort and minimize motion. A complete imaging session took about an hour, depending on the subject‐determined length of inter‐block breaks.

Analysis was performed with the Statistical Parametric Mapping toolbox (SPM‐12) on MATLAB R2018a (Penny et al., 2011). During data preprocessing, images were first corrected for slice acquisition delay and then spatially realigned to correct for head movements. Co‐registration was then performed in reference to the T1‐weighted structural image, followed by normalization to the T1 template of the Montreal Neurological Institute (MNI), with parameters derived individually from nonlinear normalization of grey‐matter T1 images. All images were then smoothed with a Gaussian kernel of FWHM = 8 mm.

### MRI analysis

A general linear model including periods of visual stimuli presentation (i.e., 1–4 s after phase onset) in each of four different phases was established. To investigate the reference memory component involved in the reproduction task, a memory component was added to the GLM model, spanning the 8‐s interval between visual object offset in EP and the cue onset in RP. The subtraction method was applied to disentangle each component. Invalid trials were excluded from the GLM model. The analysis was performed first on the individual level, resulting in an individual T‐statistic map for each comparison of interest; group analysis was then performed for each of these comparisons. Results were visualized with the help of the xjview toolbox for MATLAB (https://www.alivelearn.net/xjview) based on Automated Anatomical Labeling (http://www.gin.cnrs.fr/AAL-339) atlas.

### ROI analysis

To further investigate the temporal envelope of BOLD signal related to different cognitive processes, region of interest (ROI) analysis was performed in a set of regions derived from the group‐level simple contrasts. Spherical masks were applied for each ROI, centered at the peak voxel of the simple contrasts. The radius of each sphere was determined by the cluster size. BOLD signals were extracted with the help of the MarsBaR toolbox for MATLAB (Brett et al., 2002). The hemodynamic function (HRF) of each ROI was obtained first on the individual level, and then averaged across subjects and plotted with the between‐subject variance. All HRFs were baseline‐corrected to the average BOLD signal during the visual control phase (30–40 s after trial onset). Predicted positions of the peaks were indicated by vertical dash lines, with 6 s delay following the end of stimulus display (10 s from phase onset).

### Temporal cross‐correlation of regional hemodynamic function

Trial averaged hemodynamic functions (HDFs) for each subject were then fed into an interregional temporal cross‐correlation analysis to investigate the connectivity pattern. The HDFs were divided into four segments, corresponding to the delayed hemodynamic response for four task phases (start at 6, 18, 30, and 42 s after trial onset), resulting in a cross‐correlation function for each ROI pair. A quadratic function was fitted with the highest three points around the peak in estimation for the inter‐regional lag. A heatmap was created to visualize the inter‐regional lag between selected ROIs during each of the task phases.

## RESULTS

### Behavior verification of duration‐reproduction task

To better disentangle the brain activities related to encoding, working memory, and retrieval of interval duration, and for the sake of better signal‐to‐noise ratio, a longer inter‐stimuli interval (ISI) is required in functional imaging. Considering the relatively long TR of fMRI scanning and slow decay rate of the BOLD signal, the ISI was extended up to several seconds, which is much longer than what is usually implemented.

A behavioral pilot experiment was conducted in search of an optimal length of ISI as well as reaffirming the temporal reproduction accuracy of the 3‐s sample interval. The uniqueness of the 2–3‐s interval presumably represents a borderline of the logistic capacity of timing as it is observed in many cognitive processes (Bao et al., 2015; Chen et al., 2020; Pöppel, 1997; Pöppel, 2009; Wang et al., 2016; Yu & Bao, 2020; Zhao et al., 2018). Since our focus is on the high‐level semantic aspect of the timing function, it is reasonable to keep the temporal framework of neural processing in its optimal range to minimize the noise of low‐level processing. The pilot task design closely imitated the scanning experiment, except that the ISIs were randomly chosen from either 4.5 s or 9.0 s for each block, and the sample duration from four conditions (1.5 s, 3 s, 4.5 s, or 6 s) randomized within‐block.

Trials were excluded from analysis if the reproduced durations exceeded 3 standard deviations from the mean. A 2(ISI) × 4(Sample Duration) repeated‐measure analysis of variance found neither significant interaction effect (*p* > .1) nor significant main effect of ISI (*p* > .1); only main effect of sample duration (*p* < .001, Figure 1B). The result indicated that the reproduction performance was not altered by a longer ISI (9 s), leading to the implementation of a similar ISI during scanning (8 s). The reproduced durations for each sample duration in the long ISI condition are highly consistent with previous studies with a shorter ISI (Pöppel, 1972; Ulbrich et al., 2007).

### Performance in duration‐reproduction and motor control task

During scanning, participants were asked to reproduce a duration to match the sample (Figure 1A). Reproduced durations of all 15 participants in the scanner were analyzed: subject means ranged from 2.42 s to 3.54 s. Among the participants, reproduced durations of three subjects were not significantly variated from the sample (*p* > .05), two subjects over‐reproduced (i.e., reproduced duration >3 s, *p* < .001), while the other 10 under‐reproduced (*p* < .005). Even though identical sample duration was implemented in all trials without being explicitly indicated, most of the participants reported variations in the perceived duration during a short post‐experiment interview. Group mean of reproduced duration = 2.775 s (95% CI, [2.628, 2.921]); one‐sample *T*‐test revealed that the reproduced duration was significantly smaller than the sample duration of 3 s (*p* = .003, one‐tailed; see Table 1). Considering that the difference between reproduced duration and the sample (Mean = 0.225 s) was much smaller than the sampling rate of fMRI (TR = 2 s), this did not alter the temporal envelope of BOLD signals remarkably. Therefore, all following analyses were performed pooling over all valid trials.

**TABLE 1:** Group‐mean and 95% confidence interval (CI) of reproduced duration in Reproduction Phase, and simple reaction time in Motor Control Phase.

	Mean	*SD*	95% CI
Reproduced duration (s)	2.775	0.265	[2.628, 2.921]
Simple reaction time (s)	0.346	0.069	[0.311, 0.381]

In the motor control phase of the task, participants were asked to make a simple reaction to the offset of the visual stimulus (see Methods). The mean reaction time (RT) of participants ranged from 0.253 to 0.515 s; group mean = 0.346 s (95% CI, [0.311, 0.381], Table 1). Since the fluctuation of RT is also much smaller than the TR, the following analyses were performed pooling over trials with different RT.

### Timing and motor preparation involve partially overlapped response

Taking together the two additional control tasks, the behavior paradigm can be split into four task phases: the Encoding Phase (EP), when subjects were presented with a 3‐s visual stimulus; the Reproduction Phase (RP), when subjects were required to terminate another stimulus when its duration subjectively matched the sample; the Visual Control (VC) phase, when the same 3‐s visual stimulus was just passively viewed; and the Motor Control (MC) phase, when subjects were required to press the key (identical motor response as the RP) following the removal of visual stimuli. Therefore, each trial can be evenly divided into four task epochs with identical lengths (12 s).

**FIGURE 2:**
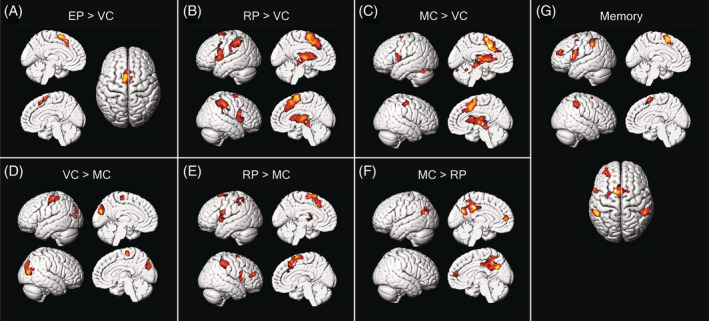
Rendered view of results displaying contrasts of interest. The period with visual stimulus display in each of the four task phases was included in the design matrix together with a memory component in between the encoding and reproduction. The design matrix was then convolved with the hemodynamic function, resulting in a GML model. Voxel‐wise comparisons between the regressors of interest led to the activity‐contrast map as depicted above. Supplementary motor area (SMA) activation was observed in both timing phases (Encoding Phase [EP] > Visual Control [VC] and Reproduction Phase [RP] > VC) as well as in the motor control and during the memory delay. Thalamus and basal ganglion structures (e.g., putamen) were activated only in the two phases containing a motor response (RP > VC and Motor Control [MC] > VC). In addition, a frontoparietal circuit including the left dorsolateral prefrontal cortex and bilateral intraparietal sulcus structures was specifically activated during the memory phase. The significant level was set at cluster‐level *p*(familywise error) < .05.

For the first part of fMRI data analysis, simple contrasts were performed with a general linear model (GLM) on the whole‐brain activation maps for the period of stimuli presentation across four task phases (Figure 2 and Table 2). Since overlapping compositions of cognitive processes were present in these four phases, active timing and time‐irrelevant activities can be disentangled with psychological subtraction: comparison between EP and VC reveals timing‐only activities free from contamination by motor components; while comparing RP with VC reveals duration working memory, decision‐making, and motion preparation activities in addition to those specific for timing. Comparison between MC and VC was also performed in search of motor‐specific components.

**TABLE 2:** Clusters with FWE corrected significance in different comparisons between task phases.

Comparison	Peak coordinates			*p* (FWE_cluster_)	Sub‐region
	x	y	z		
EP > VC	−10	−2	64	0.002	SMA L&R Medial Superior Frontal Gyrus L
RP > VC	−10	−14	4	0.000	Thalamus L&R Insula L&R Inferior Frontal Operculum L&R Putamen L&R Precentral L&R Rolandic Operculum L&R Caudate L Pallidum L Inferior Frontal Triangularis L&R
	50	−32	48	0.000	Inferior Parietal R Supramarginal Gyrus R Postcentral R Superior Parietal R
	−46	−42	48	0.000	Inferior Parietal Gyrus L Supramarginal L Postcentral L
	−8	−6	64	0.000	SMA L&R Mid‐Cingulate Cortex L&R Medial Superior Frontal Gyrus L&R Superior Frontal Gyrus L&R
MC > VC	−34	−62	−28	0.010	Cerebellum L
	−8	−14	−2	0.000	Thalamus L&R Putamen L&R Caudate L&R Insula L&R Inferior Frontal Triangularis L Pallidum L&R
	−46	2	12	0.015	Rolandic Operculum L Inferior Frontal Operculum L Precentral L Insula L
	−8	0	62	0.000	SMA L&R Mid‐Cingulate Cortex L&R Medial Superior Frontal Gyrus L Superior Frontal Gyrus L Anterior Cingulate L
	42	−22	50	0.013	Precentral R Postcentral R
RP > MC	−38	8	10	0.001	Insula L Putamen L Inferior Frontal Operculum L Pallidum L
	54	12	26	0.026	Inferior Frontal Operculum R
	−52	0	24	0.029	Precentral L Inferior Frontal Operculum L
	42	44	20	0.030	Middle Frontal Gyrus R
	−4	14	68	0.000	SMA L&R Medial Superior Frontal Gyrus L
	54	−30	54	0.000	Inferior Parietal R Supramarginal R Postcentral R Superior Parietal R
	−50	−42	62	0.042	Inferior Parietal L
Memory	−14	10	60	0.000	SMA L&R Inferior Frontal Operculum L Precentral L Superior Frontal Gyrus L Middle Frontal Gyrus L Insula L
	−26	40	26	0.028	Middle Frontal Gyrus L
	54	−36	50	0.005	Inferior Parietal Gyrus R Supramarginal R
	−56	−38	48	0.007	Inferior Parietal Gyrus L

*Note*: 3D coordinates of the peak voxel, statistical significance, and subregions of each cluster are reported. Only subregions with voxel size > 30 were included. Abbreviations: EP, encoding Phase; FWE, familywise error; L, left; MC, Motor Control; R, right; RP, Reproduction Phase; SMA, supplementary motor area.

Subtracting visual components from the encoding Phase (i.e., EP > VC) resulted in significantly activated clusters mainly in the SMA, together with a small proportion of the medial superior frontal gyrus (Table 2; see Figure 2A for rendered view on cortical templates). Reverse comparison VC > EP found no significant result.

Comparison RP > VC revealed a similar cluster at SMA and medial superior frontal gyrus, as well as additional clusters spreading across bilateral thalamus, putamen, pallidum, precentral gyrus, insula, and operculum (Figure 2B). An important difference between reproduction and encoding was the additional involvement of bilateral inferior parietal lobule (IPL), including structures alongside the postcentral sulcus and intraparietal sulcus. These reproduction‐specific brain activities can be attributed to the duration working memory: maintenance of duration information (reference memory) and its utilization in anticipatory timing (executive control). Reverse comparison VC > RP revealed no significant cluster.

To control for non‐timing motor components, a comparison MC > VC was performed (Figure 2C). Significant clusters spread in a similar set of regions as in RP, including subcortical structures in bilateral thalamus, putamen, pallidum, and caudate, as well as cerebral activations in bilateral insula and SMA. Another cluster was in the superior lobes of the left cerebellum. The operculum cortex, inferior frontal lobule, and precentral gyrus were lateralized to the left hemisphere. Since no explicit timing was required in this phase of the task, these activities should be caused by non‐timing motion preparation and motor initiation. Reverse comparison VC > MC revealed significant clusters mainly in the occipital visual areas (Figure 2D).

To disentangle motor components from active timing that were intermingled during reproduction, the comparison RP > MC was also performed, revealing higher activation levels in the putamen, pallidum, insula, and precentral cortex, all lateralized to the left (Figure 2E). Operculum activation was found to be higher during reproduction in both hemispheres. SMA also displayed elevated activity, indicating its participation in both active timing and motor planning, thus its bifunctionality. The motor planning cortical loci (SMA) may have an additional function in providing conscious access to the duration of external and internal events, during both encoding and reproduction. The BG structures (caudate, pallidum, and putamen) were activated only in the reproduction and motion control phase with a stronger activity during reproduction, questioning its participation in sensory timing.

In addition to the SMA and the BG, significant clusters located in the right middle frontal gyrus and bilateral structures alongside the intraparietal sulcus were unique for duration reproduction; again, indicating their role in active and explicit timing rather than motor planning. Reverse comparison MC > RP revealed clusters in the cingulate cortex and left medial superior frontal gyrus (Figure 2F).

### Duration reference memory located in the frontoparietal circuit

To reproduce a duration in match of a previously given sample, information about the sample duration must be kept in reference memory throughout the response delay. A memory component was thus included in the GLM, starting right after the termination of visual stimuli in ER and lasted 8 s until the beginning of the RP phase. Group analysis revealed significant clusters first in a leftward lateralized cluster composed of the SMA, operculum, precentral, and insula, then in bilateral IPL at an approximately symmetric location, and in the left dorsolateral prefrontal cortex (dlPFC; Figure 2G). This inter‐stimulus sustained activity features the engagement of these areas in maintaining the reference memory of sample duration.

### Hemodynamic function differs between timing and motor activities

Since the GLM model provides a trend‐line analysis between the observed signal and the pre‐hoc defined task design matrix, the reliability of the interpretation depends on the neuropsychologic understanding of the task itself. However, in a timing task where time itself is the information to be encoded, it is unknown what cognitive progress is taking place when. Therefore, the task design matrix of the GLM model is based on hypothetical prior assumptions about the temporal profile of cognitive processes. From a statistical perspective, the results cannot be more reliable than the assumptions, neither can they justify these assumptions. A data‐driven, hypothesis‐free approach is preferential in this case. We then took a data‐driven approach by comparing the temporal envelope of BOLD signal across different loci. Our behavior task was designed in a way that the BOLD signal sampling can be temporally aligned across all the trials. All four task epochs were extended to 12 s, corresponding to six sampling points. The whole trial added up to 48 s or 24 sampling‐point lengths of the BOLD envelope. Overlapping between the BOLD signal of adjacent events is optimally balanced between each phase of the task. The mean BOLD envelope is thus a reliable approximate of the task‐evoked HDFs in each ROI (Table 3).

**TABLE 3:** Spherical ROIs derived from significant clusters in whole‐brain level comparisons.

Location in the brain	Contrasts of identification	Coordinate of center			Radius (mm)	Peak *T* value	Voxel size
		X	Y	Z			
SMA L	EP > VC	−10	−2	64	4	6.03	33
SMA R	RP > MC	4	16	56	4	6.33	33
Thalamus L	RP > VC	−10	−14	4	6	8.59	123
Thalamus R	RP > VC	14	−18	10	6	7.05	123
Putamen L	RP > MC	−24	0	14	4	5.15	33
Putamen R	RP > MC^a^	24	0	14	4	(5.15)	33
Pallidum L	RP > MC	−18	−6	2	2	5.03	7
Pallidum R	RP > MC^a^	18	−6	2	2	(5.03)	7
Insula L	RP > MC^a^	−32	18	10	4	(8.12)	33
Insula R	RP > MC	32	18	10	4	8.12	33
Inferior Frontal Operculum L	RP > MC	−38	8	10	4	5.99	33
Inferior Frontal Operculum R	RP > MC	54	12	26	4	6.28	33
Inferior Parietal L	RP > MC	−48	−42	48	4	5.69	33
Inferior Parietal R	RP > MC	54	−30	54	4	6.91	33
Supramarginal L	RP > VC	−56	−24	24	4	5.79	33
Supramarginal R	RP > VC	50	−32	48	4	7.20	33
Middle Frontal L^a^	RP > MC^a^	−42	44	20	4	(5.40)	33
Middle Frontal L	Memory	−26	40	26	4	6.97	33
Middle Frontal R	RP > MC	42	44	20	4	5.40	33

*Note*: The center coordinate was defined by peak voxels within the clusters of interest. Abbreviations: EP, Encoding Phase; L, left; MC, Motor Control; R, right; RP, Reproduction Phase; SMA, supplementary motor area.

^a^ Marks those ROIs derived from its contralateral analog by mirroring the location, their peak *T* values were taken from their contralateral ROI and labeled with brackets.

**FIGURE 3:**
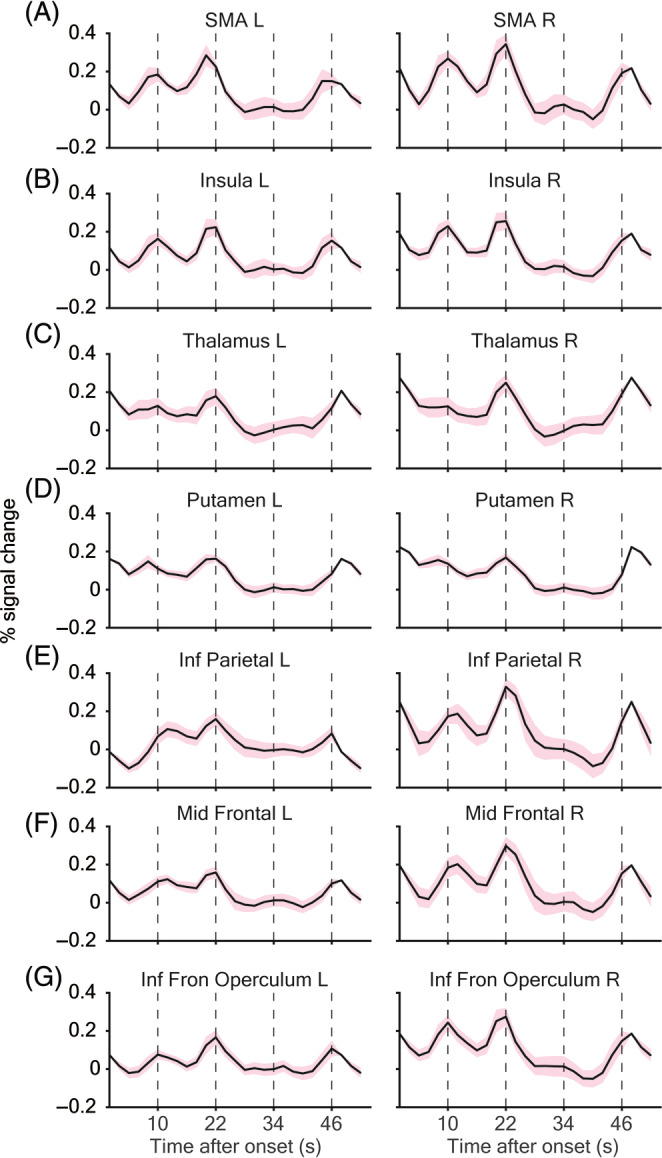
Trial average hemodynamic functions of interval‐timing‐related brain regions. Baseline was defined as the average signal strength during visual control. Paired *T*‐test was performed on the differences from baseline (red: significantly higher; green: significantly lower). Timing and motor‐related activities were observed in the (A) supplementary motor area (SMA), (B) insula, (C) thalamus, (D) the putamen, and (G) the inferior frontal operculum. An activation plateau was observed in the (E) inferior parietal lobe and (F) the middle frontal gyrus, both lateralized to the left hemisphere, probably related to the duration working memory.

In bilateral SMA (Figure 3A), the HDF showed three peaks following the encoding, reproduction, and motor control phase. Interestingly, only in the right SMA, the last peak of HDF during motor control came later than predicted and its left‐hemisphere counterpart (estimated with 6 s offset, see Methods). Considering the delay by reaction time and the left‐hand response, the delayed activity of right SMA might be better explained by the motor response. In addition, the peak amplitude following reproduction was higher than both encoding and motor control phase (paired *t*‐test, all with *p* < .001), confirming the simple contrast between RP > MC. This again supports the bifunctionality of SMA, with additive BOLD signals when both functionalities are called.

A similar time course of HDF was observed in the insula (Figure 3B), another structure previously suspected for time‐encoding (Kosillo & Smith, 2010; Wittmann et al., 2010). Signal was elevated in bilateral insula during encoding, reproduction, and motor control. A delayed peak was observed in the right insula during motor control, but not in the encoding and reproduction phases.

The HDF in the thalamus (Figure 3C) suggests that its function might be more motor‐related, with peaks only in the two phases with a motor response (i.e., reproduction and motor control). It is thus consistent with the results of simple comparisons. The right thalamus ROI revealed more dramatic signal fluctuation compared to the left side, in line with the left‐handed motor response.

The BOLD signal changes during the timing task in BG structure putamen (Figure 3D), on the other hand, were smaller in size compared to other ROIs. The signal remained high throughout the first two phases (encoding, reproduction compared to visual control), as well as during motor control, but was delayed and much steeper. It might indicate a different hemodynamic response while participating in motion generation as compared to explicit timing.

In the IPL (Figure 3E), the HDF followed a similar pattern like the SMA, with three peaks in encoding, reproduction, and motor control, and the highest response was reached during reproduction. Interestingly, a plateau of activity between encoding and reproduction was observed only in the left IPL, probably related to the maintenance of duration reference memory. Although activities in bilateral IPL were both significantly altered by the task, it seemed that the reference memory of duration information was leftward lateralized as suggested by its activity pattern.

A similar pattern as IPL was found in the middle frontal gyrus (MFG; Figure 3F), with a more prominent signal fluctuation in the right hemisphere and a plateau‐like activity in the left. Concerning results of simple contrasts that the left MFG was found significant during the memory phase and the right MFG significantly activated during reproduction, bilateral MFG might play a different role in duration reference memory (left) and executive control (right).

In contrast, no such plateau‐like activity was found in the left inferior frontal operculum (Figure 3G), another significantly activated region in both the reproduction and memory phase. It also illustrates that comparing the temporal envelope of the BOLD signal can reveal more than the common hypothesis‐driven way.

### Direction of functional connectivity differs between timing and motor activities

**FIGURE 4:**
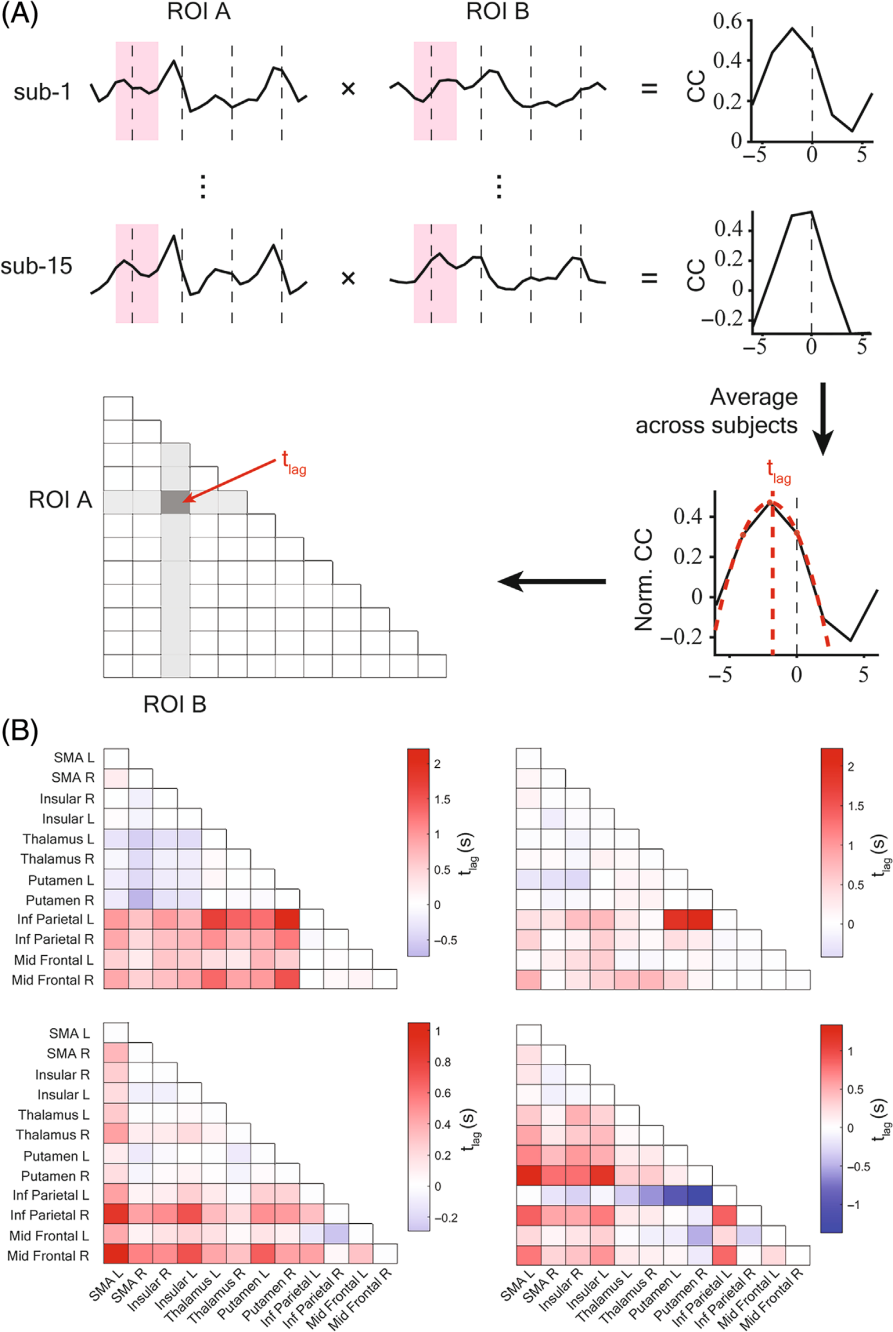
Inter‐regional cross‐correlation of hemodynamic function. (A) The trial average hemodynamic function (HDF) of each region of interest (ROI) was first obtained for each individual and then segmented into four phases. Cross‐correlation functions were calculated for each pair of ROIs and then averaged across subjects. The temporal lag between each pair of ROIs was estimated by fitting a quadratic function to the highest three data points of the cross‐correlation function. (B) Inter‐regional time lag matrix for each task phase. A negative value indicates that the region specified on the Y‐axis leads the region on the X‐axis, while a positive value indicates the X‐labeled region leading the Y‐labeled region. In the Encoding Phase (EP), thalamus and putamen activities led the SMA and insula, while the relationship was partially reversed during the Reproduction Phase (RP), in accordance with the direction of information flow.

To further investigate the strength and direction of inter‐regional interaction, a cross‐correlation analysis was performed on the HDF for each pair of ROIs within the four task epochs. The peak of activity was estimated by fitting a quadratic function to the more sparsely sampled data. This allows interpolation beyond the 2‐s sampling rate of the fMRI (see Methods and Figure 4A). Dramatic changes in the direction of inter‐regional correlation were observed in the inter‐regional time lag matrix (Figure 4B): during encoding, BOLD signals in the putamen and the thalamus led the SMA and the insula, which then led the frontoparietal circuit. While during reproduction and motor control this relation was partially reversed, that is, the SMA and insula signals led the putamen, which then led the thalamus. The temporal sequence suggests the direction of information flow through the hierarchy: duration information is transferred in the bottom‐up direction from subcortical regions in the BG and thalamus to cortical regions in the SMA and insula; while the same pathway is re‐used but in a reversed direction down the hierarchy during motor output.

For higher associative cortical regions, such as the middle frontal gyrus and the inferior parietal lobe, the parietal ROIs slightly led the frontal ROIs during encoding, which was remarkably reversed during reproduction, with the left middle frontal region leading the parietal ROIs. It should also be noted that during the visual control phase when significant activity is often missing, the interpolation method we used for inter‐regional temporal lag estimation is not as reliable as the other three phases.

## DISCUSSION

Disentangling parallel cognitive processes is the core challenge in neurocognitive research on timing. All physical events, including those in the brain, take a certain interval. The most common methodology in analyzing neurophysiological data (BOLD fMRI, electrical signals in intra−/extra‐cellular recording, EEG etc.) is to correlate the recorded time series with the temporal profile of external or internal events. However, interpretation of this temporal correlation becomes problematic when several events temporally coincide. This problem is especially eminent when the duration of time itself is the input to the neuronal system, as in timing tasks.

In previous imaging studies, researchers either looked for accumulating signals or simply compared brain activities between a timing task and a non‐timing task in a block design. However, both approaches fail to address whether the observed activities are truly relevant for dedicated timing in the brain. The same problem persists even when temporal information can be post‐hoc decoded from neural signals (Bruce et al., 2021; Cueva et al., 2020). There is no guarantee that the embedded decodable time is utilized in perception or motor timing. Distinction should be made between time as a *logistic function* of the neural system that acts as the context of neural processing (Pöppel, 2009) and as a *semantic function* that provides the duration as a cognitive content.

One historical approach to disentangle different cognitive processes in intermixed data is the psychological subtraction method invented by Donders (Bao et al., 2016; Donders, 1969). The subtraction method was first implemented on reaction time, following the idea of sequential processing, but has been frequently revisited in modern neuroscience. Contrast between whole‐brain activity maps of different tasks is a typical example of subtraction. It has the potential to differentiate activities even when they share the same time course.

The duration reproduction paradigm is widely used in timing research partially due to its rich cognitive composition. It consists of a near‐complete spectrogram of cognitive components: encoding, memory, and retrieval of duration information, for both external sensory input and prospective motion planning. However, its complexity also adds to the difficulty of interpreting the data. Linking any activity that is temporally evolving with the timing mechanism risks contamination from non‐timing parallel components. In this study, we tried to dissect the duration‐reproduction task by comparing the hemodynamic function of the timing task with two control tasks, in both hypothesis‐driven and data‐driven ways.

The BG–SMA circuit has been repeatedly reported in various timing tasks and has thus been suspected as the neural substrate of dedicated timing (Casini & Vidal, 2011; Ferrandez et al., 2003; Kotz et al., 2016). However, considering the BG's more general role in goal‐directed, task‐context‐sensitive behavior (Haber, 2003; Korb et al., 2017), it is questionable whether their neurophysiological responses represent merely a logistic function of time that coordinates activities across the cortex. Our result shows that the SMA is involved in both timing and motor planning, with an additive BOLD response when both cognitive components are present during reproduction. Similar signatures of activity were observed in the insula but turned out to be statistically insignificant during encoding. Comparatively, the BOLD signal in part of the dorsal striatum, the putamen, was subtle during encoding and reproduction but had a sharp response during motor control. However, given its elevated response throughout EP and RP compared to VC, the putamen may also be bifunctional, only less specific during timing than its well‐known function in motion initiation (Turner & Desmurget, 2010). We also found in temporal cross‐correlation that the direction of connectivity went from BG to SMA during encoding but from SMA to BG and thalamus during reproduction, suggesting some information might have been passed from BG to SMA during encoding. We propose that the additive hemodynamic response in the SMA may result from distinct neural populations and independent microscopic organizations for the motor and timing functions, while the relatively flat fluctuation in the BG may represent overlapping and multifunctional neural populations. In that case, representation of time is generated in the cortex by interpreting the subcortical inputs differently in a goal‐directed manner, while the temporal coordination in the BG is logistic and not semantic function by itself.

The most persuasive evidence on timing in the BG comes from clinical research (Dušek et al., 2012; Högl et al., 2014) and animal studies (De Corte et al., 2019; Kononowicz & van Rijn, 2014). The midbrain dopaminergic input to the striatum was suspected to be relevant (Mello et al., 2015; Parker et al., 2015; Soares et al., 2016). However, in these studies, the timing function is evaluated on the net output of the whole system; it might be that the subcortical dopamine dynamic influences the timing behavior via acting on the logistic temporal function (e.g., coordinating the cortical activities) instead of the semantic function, which is located at a higher hierarchy in the cortex (Bao et al., 2015). In addition, the length of duration also matters: shorter durations (sub‐second to around 2 s) can be automatically tracked by the SMA‐BG motor circuit, while longer durations (more than 2 s) require additional abstraction and “counting” via the frontoparietal circuit (Morillon et al., 2009; Pöppel, 1997).

In addition to the SMA, a working memory network was observed in the dlPFC and bilateral structures alongside the intraparietal sulcus. Noticeably, the left dlPFC leads the parietal regions during reproduction, indicating the direction of executive control. The dlPFC is critical for working memory manipulations and computations, as shown in lesion (Barbey et al., 2013) and imaging studies (Olesen et al., 2004). The intraparietal sulcus is known to be important for working memory of not only duration but also other domains of magnitudes, as so‐called “general magnitude system” or “approximate number system” (Bueti & Walsh, 2009; Nieder et al., 2006; Walsh, 2003). Evidence supporting duration representation in the general magnitude system comes from clinical observations of the deficit timing function in developmental dyscalculia patients (Skagerlund & Träff, 2014); imaging studies showing common activation of inferior parietal lobe by number, spatial magnitudes, and duration (Bueti & Walsh, 2009; Sokolowski et al., 2017); cross‐modality transferring effects under training or brain stimulation (Cappelletti et al., 2013; Lourenco et al., 2016); as well as developmental and non‐human primate studies (Jacob et al., 2012; Nieder, 2016). This frontoparietal circuit abstracts the duration of several seconds into a magnitude representation that allows for active manipulation, such as comparison between memory items.

The two different cortical systems related to timing, along with the supporting subcortical structures, may perform a different level of abstraction and thus compose a hierarchy of functions for duration processing. In subcortical structures, time is not explicitly encoded, but rather as a logistic function that temporally synchronizes different neural subsystems in the brain. The same network and population of BG neurons might broadcast to cortical downstream areas of all functions, only being interpreted differently in a task‐dependent manner by the receiver. In the first‐order cortical region for timing (i.e., SMA), these subcortical inputs are integrated in a way that duration can be actively measured, thus translating the logistic time into a form of content (i.e., the semantic aspect of time). This first level of abstraction can be realized via firing‐rate modulation (“ramping” activities) in a subpopulation of neurons (Bruce et al., 2021; Cueva et al., 2020). The increased neuronal activity might in turn affect the energy consumption in the region and thus lead to the observed climbing BOLD signal. Following this first‐order encoding, more abstract representation is created in the frontoparietal circuit as a domain of magnitude, taking the form of populational representation (Tudusciuc & Nieder, 2007). This allows for complicated cognitive manipulation and is more stable and energy efficient. A small number of neural subgroups were sufficient to code the duration without increasing their energy consumption over time, which would explain the plateau of BOLD signal in SMA.

Taken together, by comparing BOLD signals between different tasks in multiple brain areas, we were able to disentangle different cognitive components of the duration‐reproduction task. Based on differentiating the logistic function and the semantic function of time, we attribute the devoted timing mechanism to the cortex. More specifically, two distinct cortical circuits were involved: the SMA displayed additive BOLD signal for both timing and motor planning; and a frontoparietal circuit with typical delayed working‐memory activities. The plateau of BOLD signals in these working memory regions suggests different coding strategy and different levels of abstraction for duration information. Activities in the subcortical regions, such as the putamen and thalamus, are less specific. This may be due to multiplexed neuronal involvement in different functions in these subcortical regions. Thus, we propose the distinction between the subcortical logistic function of time and the cortical semantic function of time.

However, although we succeeded in attributing different processing stages to different brain loci, one should keep in mind that functional imaging catches only a summation of underlying neurocognitive and neurophysiological processes. The neural coding mechanisms underlying each level of this hierarchy remain unclear. More detailed and micro‐circuit investigations are required to justify our hypothesis. Besides, to avoid the interference by highly rhythmic noise generated by the fMRI scanner, all stimulus material in our experiment were delivered visually. Future studies should also include tasks in the auditory domain to investigate whether such timing mechanism is independent of sensory modality, thus showing a domain‐general characteristic.

## CONCLUSION

We report two core brain circuits that showed timing‐specific activity after controlling for parallel components: (1) the SMA participates in both motor planning and timing during duration reproduction, with an additive hemodynamic response; and (2) the left dlPFC and the bilateral intraparietal sulcus display activity related to working‐memory of duration information. Comparatively, subcortical structures, including the putamen and thalamus, do not differ between timing and motor‐control, and thus can be attributed to motor preparation and initiation. The temporal correlation between hemodynamic responses in ROIs confirms the expected direction of information, with SMA leading the frontoparietal circuit during encoding of duration, and with reversed sequence during reproduction. Our work supports the dissociation of two levels of abstraction during cognitive processing of temporal duration. However, due to the vague relationship between hemodynamic response and the underlying neural processes, our research cannot resolve the detailed coding mechanism in the two systems. Cellular recording methods, such as electrophysiology and calcium imaging, are needed to examine how duration was coded in these two different circuits.

## CONFLICT OF INTEREST STATEMENT

The authors declare no conflicts of interest.

## ETHICS STATEMENT

The study was approved by the Ethical Committee of the School of Psychological and Cognitive Sciences, Peking University, in agreement with the Declaration of Helsinki.
